# Four Cases of Single-Fraction Stereotactic Body Radiation Therapy for Prostate Cancer

**DOI:** 10.7759/cureus.70062

**Published:** 2024-09-23

**Authors:** Ayane Yasui, Subaru Sawayanagi, Yuki Nozawa, Daichi Sugahara, Hideomi Yamashita

**Affiliations:** 1 Department of Radiology, The University of Tokyo Hospital, Tokyo, JPN

**Keywords:** imrt, prostate cancer, single-fraction radiotherapy, single fraction stereotactic radiosurgery, stereotactic body radiotherapies, vmat

## Abstract

Hypofractionated radiotherapy for prostate cancer has been reported to date. Here, we report on four patients with prostate cancer who were treated with single-fraction stereotactic body radiation therapy. It was conducted with reference to some previous clinical trials. The median age of the patients was 76.5 years (range: 72-89 years). All except one patient with low-risk prostate cancer received androgen deprivation therapy (ADT) before irradiation. All patients received a dose of 24 Gy in one fraction using X-ray photon beams when prostate-specific antigen (PSA) fell to low levels due to ADT. After irradiation, all patients had a gradual decline in PSA, and so far none has had a PSA recurrence. Although Grade 1-2 adverse events occurred in all cases, none of the patients showed adverse events of Grade 3 or over during the observation period.

## Introduction

In the treatment of prostate cancer, hypofractionated radiotherapy is not inferior to conventional fractionated radiotherapy in terms of overall response and survival. Additionally, in hypofractionated radiation therapy, compared to conventional fractionation, the incidence of early adverse events, primarily in the genitourinary (GU) events, is increased, while the incidence of late adverse events remains similar. The rate of Grade 3 adverse events is a few percent for both methods, and Grade 4 adverse events have not been observed. Therefore, it is known that hypofractionated radiation therapy is not clearly inferior in terms of safety compared to conventional fractionation [1,2].

At the Department of Radiation Therapy of the University of Tokyo Hospital, the dose fraction of stereotactic body radiation therapy (SBRT) for prostate cancer is usually 36.25 Gy in 5 fractions, 40 Gy in 5 fractions, 42.5 Gy in 5 fractions, or 45 Gy in 5 fractions every other day. The dose was determined based on the presence of comorbidities such as diabetes and whether a SpaceOAR^TM^ (Boston Scientific, Malborough, MA) has been inserted or not. For patients who find it difficult to visit the hospital for treatment or to be admitted, a single irradiation is performed. For all patients, SpaceOAR had to be injected appropriately, which means SpaceOAR was injected in the correct position with the full volume distributed fairly evenly, based on a past randomized phase II trial [3].

## Case presentation

A total of four patients were treated for prostate cancer and received single-fraction SBRT between 2021 and 2023 in the Department of Radiation Therapy of the University of Tokyo Hospital. Table 1 shows the characteristics of the four patients.

**Table 1:** Patients' characteristics ADT, androgen deprivation therapy; iPSA, initial PSA; PSA, prostate-specific antigen. *N1: right obturator lymph node; M1: sacrum.

	Age (years)	cTNM	iPSA (ng/ml)	Gleason score	ADT (months)	PSA nadir (ng/ml)
Case A	72	cT2aN0M0	6.2	3+3 (6/20 sets)	－	0.73
Case B	89	cT3aN0M0	11.9	4+3 (6/20 sets)	3.5	0.01
Case C	72	cT2aN0M0	7.4	4+3 (5/10 sets）	3	0.03
Case D	81	cT3bN1M1*	40.5	4+5 (8/14 sets）	115	0.02

**Figure 1:**
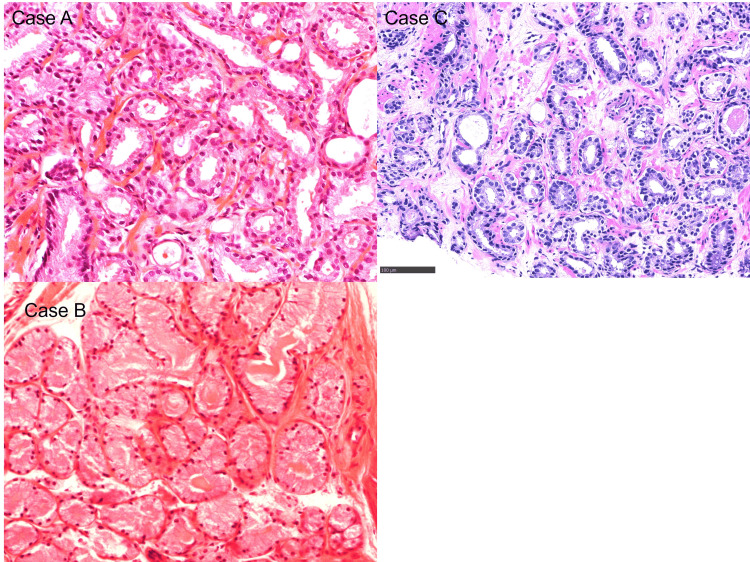
Histopathology images of needle biopsies from Cases A, B, and C. The needle biopsy of Case D was performed more than 10 years ago, and therefore, the data is no longer available. Case A, B, C: Hematoxylin and eosin staining. Atypical adenoid ducts suspicious of adenocarcinoma are seen. Histological findings of Cases A, B, and C confirm a diagnosis of prostate cancer.

The diagnosis was confirmed histologically by prostate needle biopsy, and the clinical stage was classified according to the Union for International Cancer Control 8th edition (Figure 1).

The absence of distant metastases was confirmed using positron emission tomography (PET) imaging scans and whole-body bone scans before treatment.

SpaceOAR is injected into the perirectal fat to displace the rectal wall away from the prostate in all cases before the simulation computed tomography (CT) and magnetic resonance imaging (MRI). The simulation CT and MRI with a slice thickness of 1 mm were performed in a supine position for SBRT. The patient underwent an enema and began storing urine 2 hours and 15 minutes prior to the CT and MRI scans. The simulation CT images were then co-registered with the MRI to identify the target volumes accurately and generate the treatment plan. The patient was treated on Elekta Synergy. The treatment plan was using the Monaco radiotherapy treatment planning system and the planning technique was SBRT with 6 MV or 10 MV photon beams in FFF mode. The prescribed dose was 24 Gy in one fraction on the prostate and the dose of 24 Gy was prescribed to D 95％ of the planning target volume (PTV). The gross tumor volume (GTV) was contoured in the area with high signal in diffusion-weighted imaging. The clinical target volume (CTV) was contoured involving the whole prostate and seminal vesicle (low risk: only prostate, intermediate risk: including seminal vesicles within 10 mm from prostate, high risk: including seminal vesicles within 20 mm, T3b including entire seminal vesicle). The PTV was contoured adding 5 mm to the CTV in all directions except posteriorly, where it was 3 mm. Dose constraints were based on the dose constraints of the previous phase II study [3] (Table 2).

**Table 2:** Treatment planning dose-volume constraints for single dose (24 Gy) Dose constraints were based on the dose constraints of the previous phase II study [3]. PTV, planning target volume. *DX%: Dose delivered to X% of the target volume. **D max: Maximum dose. ***D (X cc): Dose delivered to X cc of the target volume.

Dose-constrained object	The values for the dose-volume constraints	Penalties to control PTV dose coverage
PTV-D95%*	Dmax**＜107％	24 Gy
Rectal wall maximum	<95％ of the prescribed dose	<22.8 Gy
Rectal wall D (1 cc)***	<80％ of the prescribed dose	<19.2 Gy
Rectal wall D50%		<12 Gy
Urethra maximum	<95％ of the prescribed dose	<22.8 Gy
Urethra D (1 cc)	<80％ of the prescribed dose	<19.2 Gy
Bladder wall maximum	<100％ of the prescribed dose	<24 Gy
Bladder wall D (1 cc)	<95％ of the prescribed dose	<22.8 Gy
Bladder wall D50%		<12 Gy
Small/Large bowel maximum		<10/12 Gy
Urogenital diaphragm	<95％ of the prescribed dose	<22.8 Gy
Neurovascular bundles	<100％ of the prescribed dose	<24 Gy
Penile bulb	<100％ of the prescribed dose	3 cc 12 Gy
Femur head maximum		<16 Gy

There were four men with a median age of 76.5 years (range: 72-89 years). The clinical details of the four patients are displayed in Table 1. Prostate biopsies were performed, and all of them revealed prostate adenocarcinoma (Figure 1). Three patients except Case A received androgen deprivation therapy (ADT) such as degarelix 240 mg, leuprolide-SR, and first- and second-generation antiandrogens. No other sites of distant metastasis and lymph involvement were identified from the PET-CT scans, and the whole-body bone scans were negative for skeletal involvement in all patients at the start of radiotherapy.

Case A

**Figure 2:**
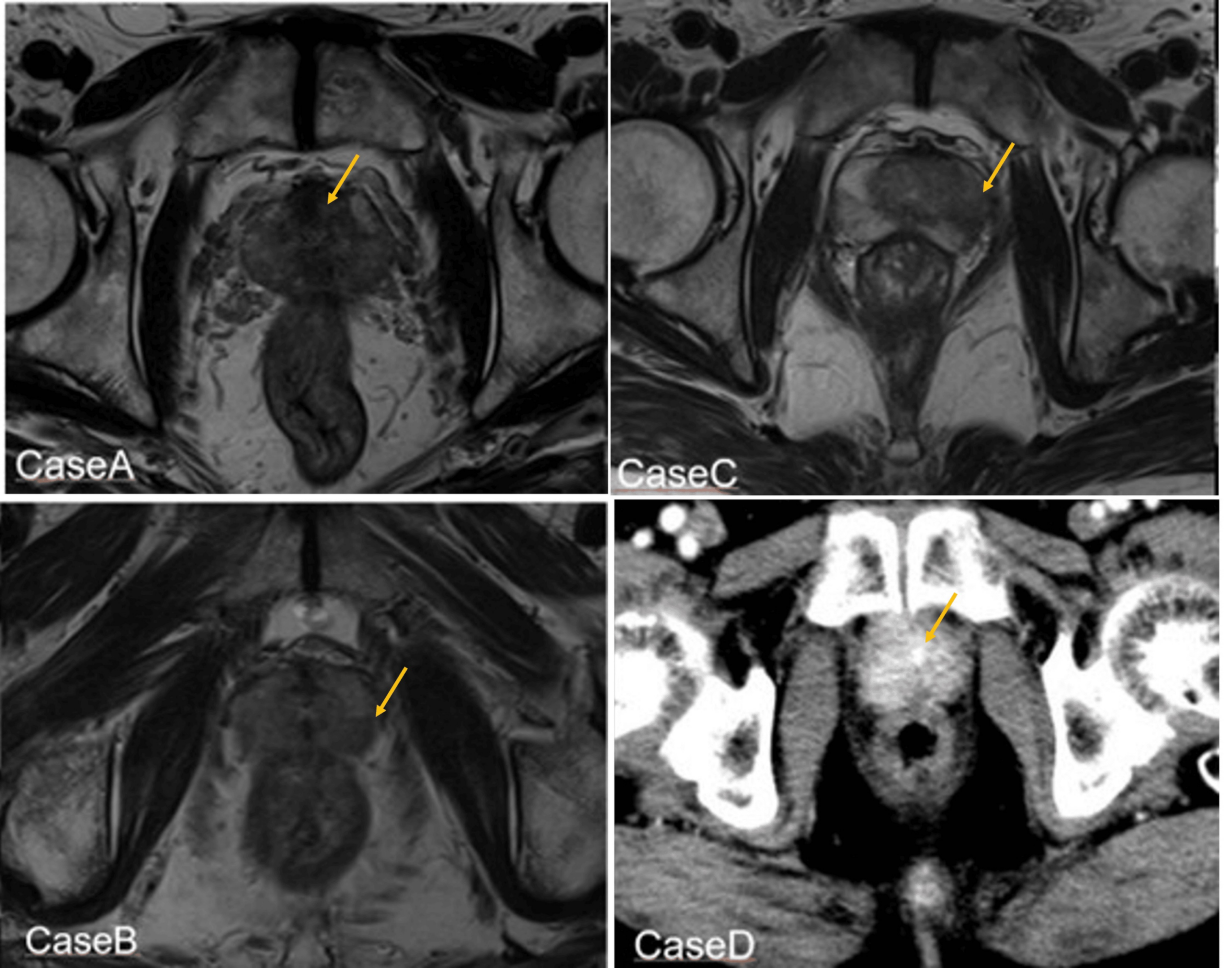
MRI images (axial) of Cases A, B, and C and contrast-enhanced CT image (axial) of Case D Prostate cancer is indicated by orange arrows. Cases A-C: At the site indicated by the arrow, T2-weighted imaging reveals an area of low signal intensity distinct from the normal structure. Case D: Contrast-enhanced CT revealed an enhancement effect in the right marginal area. CT, computed tomography; MRI, magnetic resonance imaging.

A 72-year-old man with low-risk prostate cancer (cT2aN0M0). Since he was taking anticoagulants to prevent cerebral infarction, he was referred to our department for radiotherapy. MRI revealed a 9 × 7 mm-sized prostate cancer in the left lateral transition zone (Figure 2).

Since it was difficult for him to visit the hospital five times for treatment, we decided on the treatment of single-fraction SBRT. Before treatment, SpaceOAR was injected and the insertion resulted in the creation of 47 mm (cranio-caudal) by 13.4 mm (dorso-ventral) gel-space ratio. Then the SBRT was done. The on-time of the beam was about 4 minutes.

**Figure 3:**
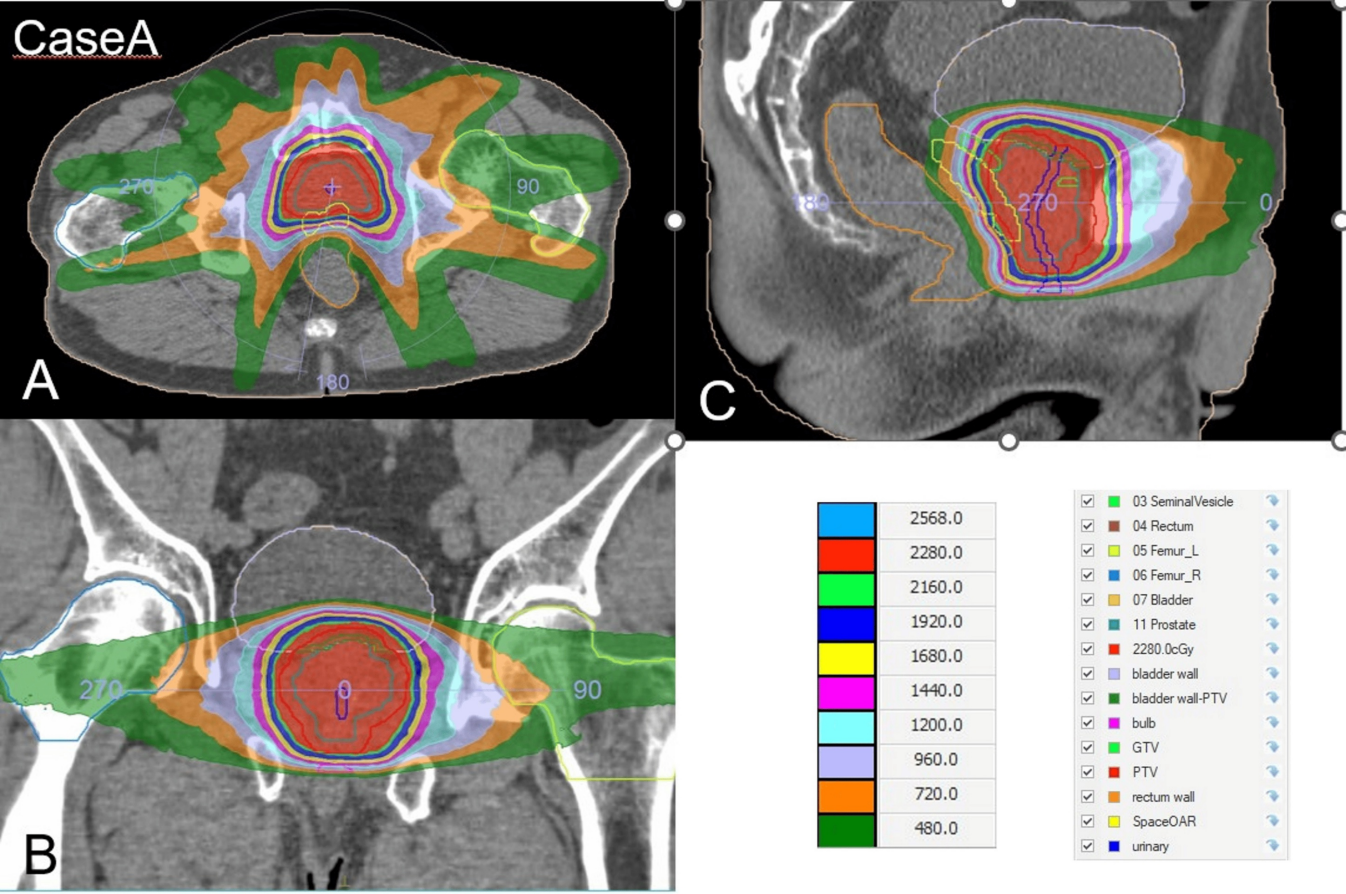
The dose distribution of Case A (A) Axial plane. (B) Coronal plane. (C) Sagittal plane. The red region indicates the 95% isodose area of the prescribed dose (24 Gy).

Figure 3 and Table 3 show the dose distribution and the OAR dose of this patient, respectively.

**Table 3:** The OAR dose of four cases. PTV, planning target volume. *DX%: dose delivered to X% of the target volume. **Dmax: maximum dose. ***D(X cc): dose delivered to X cc of the target volume. ****L: left femur; R: right femur.

Dose-constrained object	Case A	Case B	Case C	Case D
PTV-D95% (Gy)*	24.0	24.0	24.0	24.0
Rectal wall maximum (Gy)**	21.8	25.4	22.1	21.5
Rectal wall D 1 cc (Gy)***	13.4	23.6	17.1	18.8
Rectal wall D 50% (Gy)	7.5	7.5	6.6	6.6
Urethra maximum (Gy)	25.2	25.2	25.2	25.2
Urethra D 1 cc (Gy)	23.9	20.7	24.0	24.0
Bladder wall maximum (Gy)	25.5	25.8	25.2	25.2
Bladder wall D 1 cc (Gy)	24.8	25.0	24.5	24.5
Bladder wall D 50% (Gy)	1.5	0.7	1	1
Penile bulb (cc)	0.2	0.6	3.1	0.3
Femur head maximum (Gy)	L: 11.0, R: 9.7****	L: 10.8, R: 10.3	L: 10.7, R: 11.7	L: 11.4, R: 11.5

**Figure 4:**
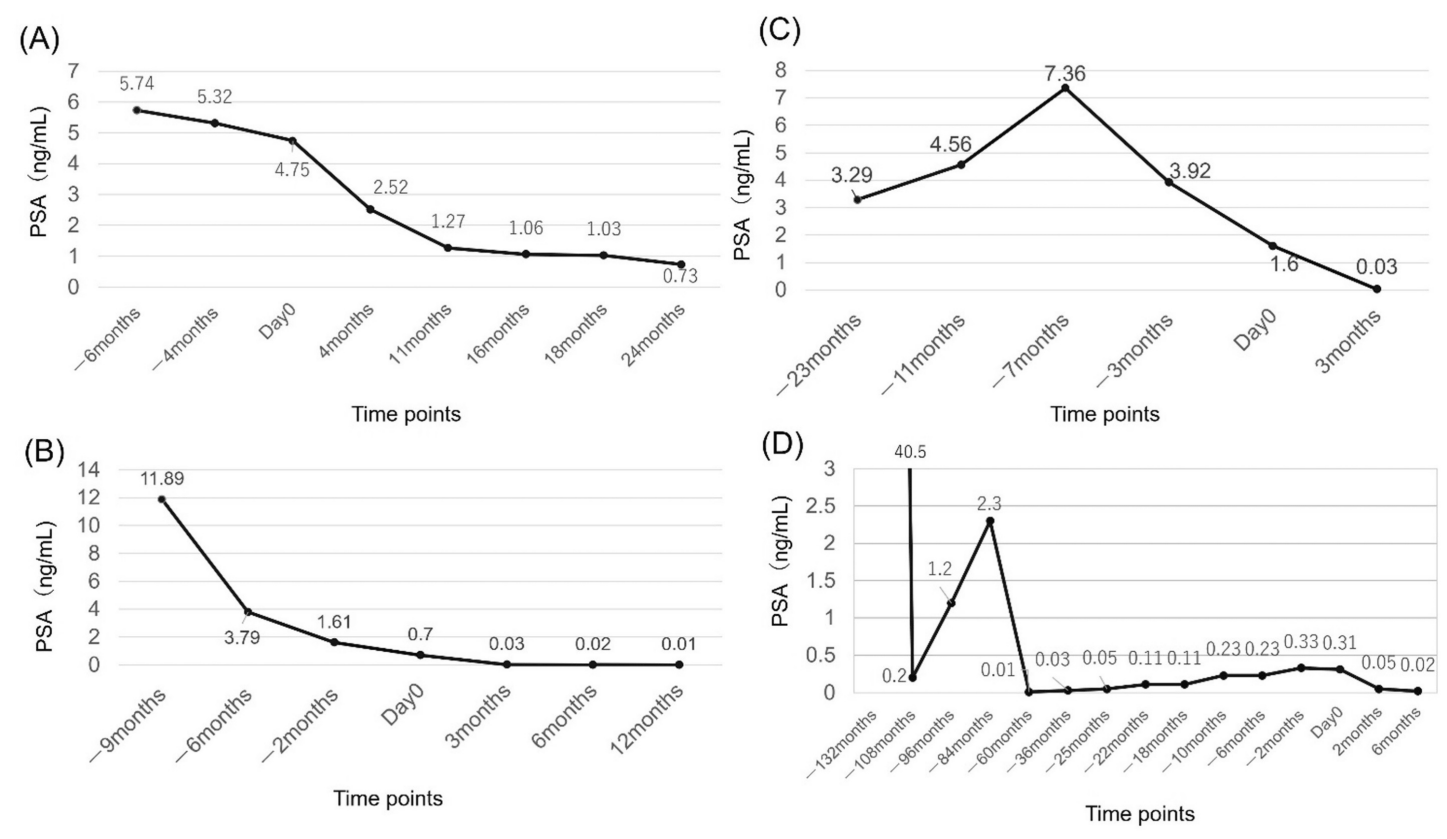
Changes in PSA levels for the four cases. (A) Case A. (B) Case B. (C) Case C. (D) Case D. The date of irradiation is set as Day 0. After irradiation, all patients had a gradual decline in PSA. PSA, prostate-specific antigen.

After the SBRT, PSA declined steadily until the two-year point after radiotherapy, and PSA remained low. At the 4-, 11-, 18-, and 24-month follow-up examinations, the PSA was 2.52, 1.27, 1.03, and 0.73 ng/mL, respectively (Figure 4).

Frequent urination temporarily worsened as an acute GU adverse event but improved to the same frequency as before radiotherapy after about 18 months. No acute or late gastrointestinal (GI) adverse events were observed.

Case B

An 89-year-old man with localized high-risk prostate cancer (cT3aN0M0, Union for International Cancer Control 8th edition). MRI revealed an 11 × 7 mm size of prostate cancer with extracapsular invasion in the marginal region of the apex of the left lobe of the prostate (Figure 2). The patient was elderly and had poor activities of daily living (ADL), which made surgery difficult. For those reasons, the patient was consulted at our department. The patient then received ADT. Before treatment, SpaceOAR was injected and the insertion resulted in the creation of 38.2 mm (cranio-caudal) by 10 mm (dorso-ventral) gel-space ratio. Then the SBRT was done. The on-time of the beam was about 7 minutes.

**Figure 5:**
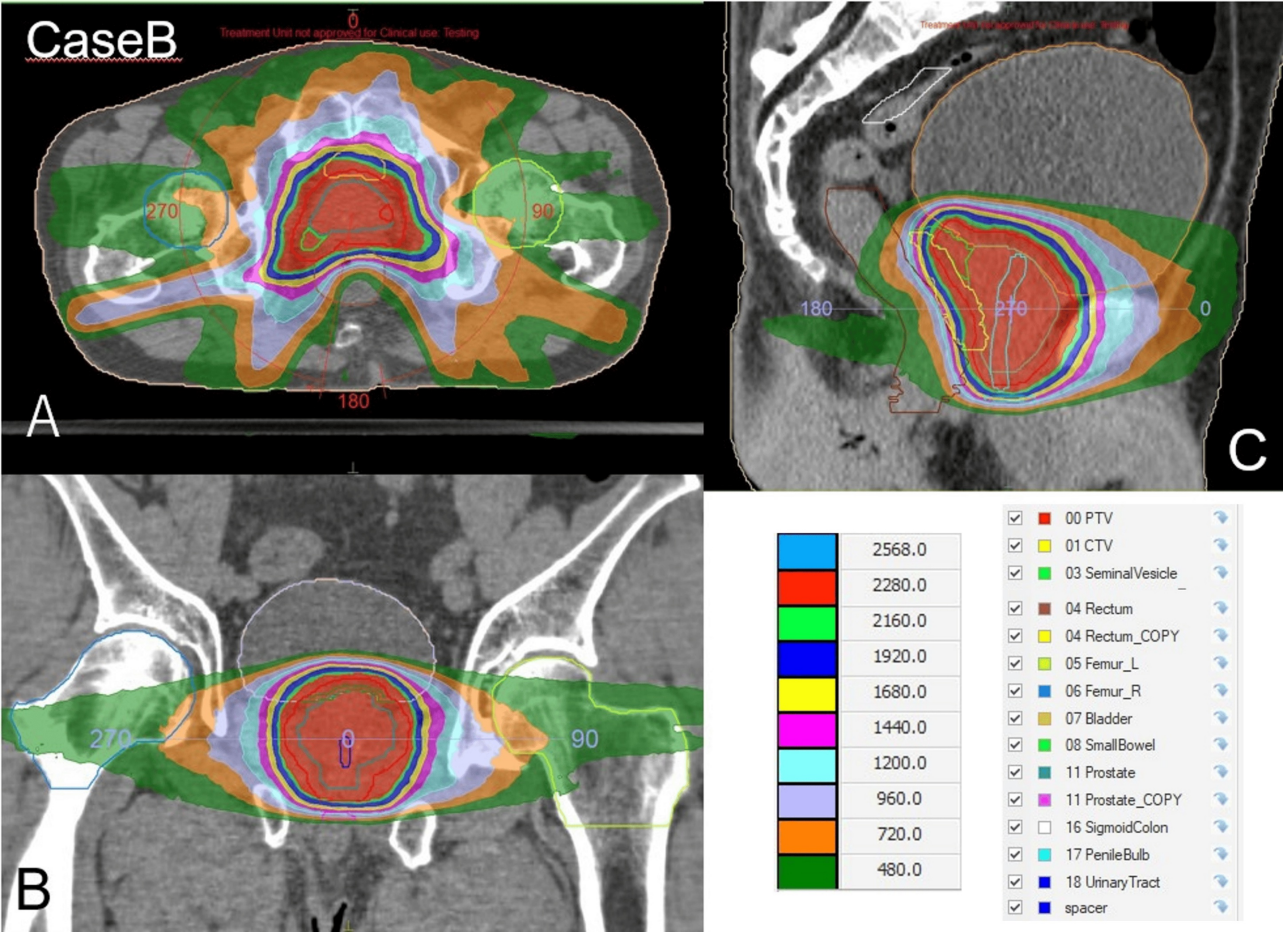
The dose distribution of Case B. (A) Axial plane. (B) Coronal plane. (C) Sagittal plane. The red region indicates the 95% isodose area of the prescribed dose (24 Gy).

Figure 5 and Table 3 show the dose distribution of the patient and the OAR dose, respectively.

Frequent urination temporarily worsened as an acute GU adverse event. After the SBRT, the level of PSA declined steadily. The PSA levels were 0.03, 0.02, and 0.01 ng/mL at the 3-, 6-, and 9-month follow-up examinations, respectively (Figure 4B). The patient did not show late toxicity.

Case C

A 72-year-old man with unfavorable intermediate-risk prostate cancer (cT2aN0M0). MRI revealed a 14 × 7 mm-sized prostate cancer in the left lateral transition zone (Figure 2). He visited our urology department and a robot-assisted laparoscopic radical prostatectomy was recommended, but he requested radiotherapy, so he visited our department. Since it was difficult for him to visit the hospital five times for treatment due to a busy work schedule, we decided on the treatment of single-fraction SBRT. Before treatment, SpaceOAR was injected and the insertion resulted in the creation of 56.5 mm (cranio-caudal) by 12.0 mm (dorso-ventral) gel-space ratio. Then the SBRT was done. The on-time of the beam was about 5 minutes.

**Figure 6:**
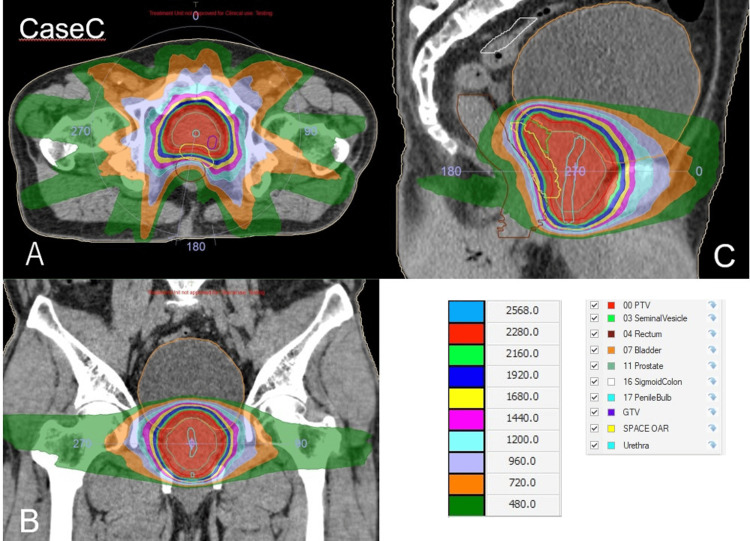
The dose distribution of Case C. (A) Axial plane. (B) Coronal plane. (C) Sagittal plane. The red region indicates the 95% isodose area of the prescribed dose (24 Gy).

Figure 6 and Table 3 show the dose distribution of the patient and the OAR dose, respectively.

After the SBRT, PSA declined, and PSA at three months after irradiation was 0.03 ng/mL (Figure 4). At three months post-irradiation, GU adverse events including decreased urinary output, urinary urgency, frequent urination, nocturia (from 0 to 2-3 times), and urinary incontinence (Grade 2) were observed. His pre-treatment IPSS score was 1, but worsened after treatment and his score at three months post-treatment was 9. No acute and late GI adverse events were observed at three months post-treatment.

Case D

An 81-year-old man with castration-resistant prostate cancer visited his local doctor and was found to have a high PSA level (40.5 ng/mL). Since then, he has undergone various ADTs for 115 months. PSA levels showed a gradual upward trend thereafter, and then contrast-enhanced CT scans were performed, which showed an area of contrast effect in the right lobe of the prostate gland and seminal vesicle (ycT2aN0M0) that was suspicious for prostate cancer (Figure 2D). The lesion was approximately 17 mm in length. PET-CT scans also showed hot spots in the area of suspected prostate cancer, but there was no evidence of distant metastasis. The patient was elderly and had poor ADL, which made surgery difficult. For these reasons, the patient chose radiation therapy and requested a treatment method with fewer clinic visits because he had spinal canal stenosis. Therefore, we decided on the treatment of single-fraction SBRT. SpaceOAR was performed after confirming the disappearance of seminal vesicle invasion by ADT. SpaceOAR was injected and the insertion resulted in the creation of 42 mm (cranio-caudal) by 16.8 mm (dorso-ventral) gel-space ratio. Then the SBRT was done. The on-time of the beam was about 7 minutes.

**Figure 7:**
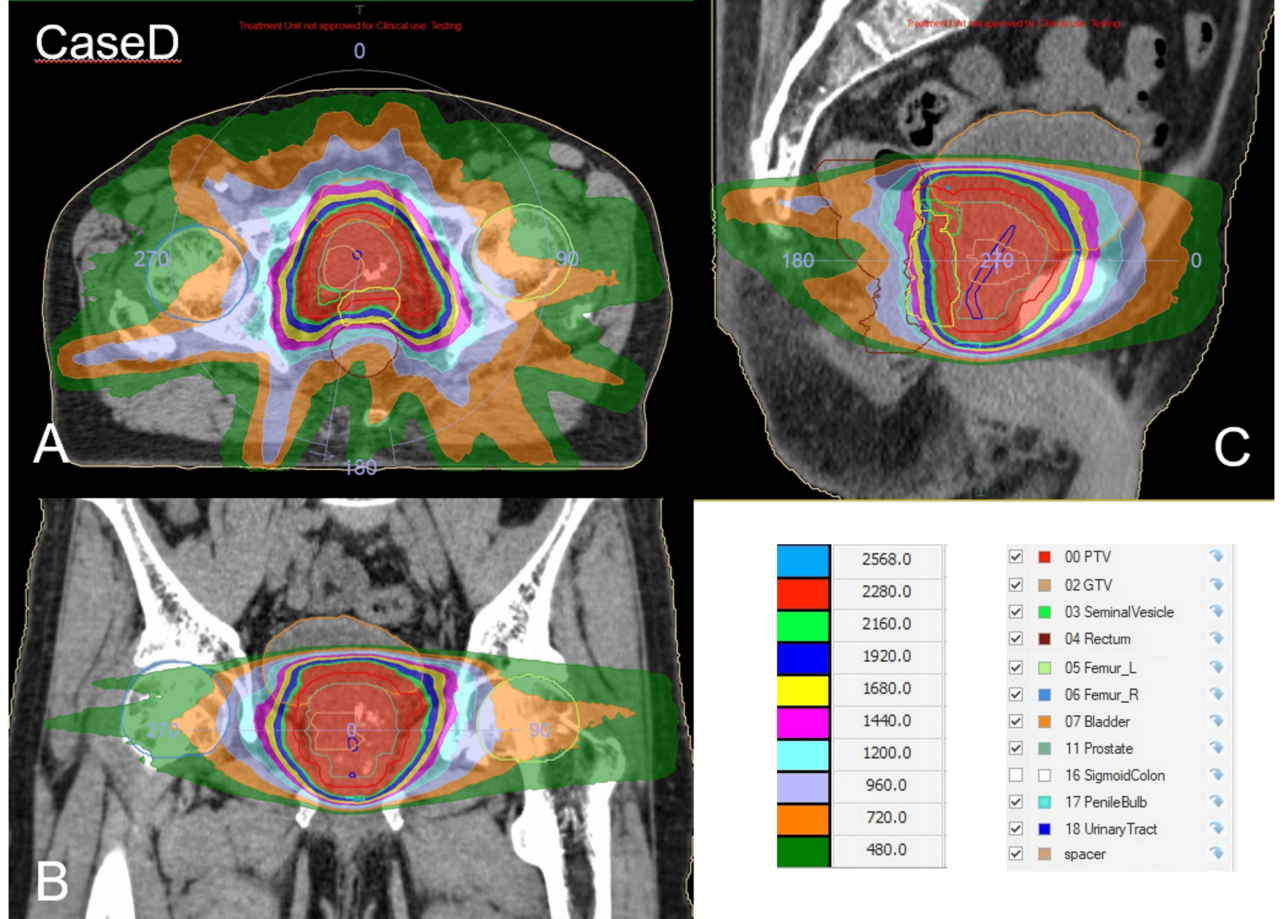
The dose distribution of Case D. (A) Axial plane. (B) Coronal plane. (C) Sagittal plane. The red region indicates the 95% isodose area of the prescribed dose (24 Gy).

Figure 7 and Table 3 show the dose distribution of the patient and the OAR dose, respectively.

After the SBRT, PSA declined and the PSA values were 0.05 and 0.02 ng/mL, respectively (Figure 4). The patient continues to receive Leuplin PRO. At three months post-irradiation, GU adverse events include decreased urinary output, urinary urgency, and nocturia (from 0 to 2-3 times). Diarrhea was observed as an acute GI adverse event, but no antidiarrheal drug was needed and symptoms improved in the third month after treatment. Mild bloody stools that did not require treatment were seen as GI adverse events in the three months after treatment.

All patients had a gradual decline in PSA after radiotherapy. In addition, no PSA recurrence and adverse events of Grade 3 or over were observed during the observation period.

## Discussion

In intermediate- to high-risk prostate cancer, hypofractionated irradiation is known to be non-inferior to conventional fractionated irradiation, with similar rates of long-term adverse events [1,2]. Ultra-high-dose single radiation therapy has demonstrated excellent long-term local tumor control rates in the treatment of oligometastatic prostate cancer, revealing the sensitivity of prostate cancer [4-6]. A single fraction of 19 Gy high-dose-rate brachytherapy is well-tolerated and has less acute and chronic GU and GI toxicity. However, the outcomes for biochemical control are not as favorable compared to fractionated irradiation [7-9]. In this study, we performed single-dose irradiation for prostate cancer based on a randomized clinical trial in which a single dose of 20 Gy or less was considered inadequate for resection of primary prostate cancer [3].

We performed single-fraction SBRT (24 Gy in one fraction) for prostate cancer referring to a study [3] that compared 9 Gy × 5 Fr SBRT with 24 Gy in one-fraction SBRT for intermediate-risk prostate cancer. In the referenced study, a rectal balloon filled with 150 cc of air was inserted to inhibit prostate movement, and a urethral catheter was used to track prostate movement during irradiation. Because these devices minimized prostate movement during irradiation, they had set GTV (prostate and seminal vesicle) = CTV and PTV = CTV + 2 mm (the surface of the OAR was reduced to 0 mm). Instead of using these instruments, we used SpaceOAR to lower the rectal dose and added a PTV margin of 3 mm (1 mm on the rectal side) compared to the reference study. In case A, the maximum rectal dose exceeded the dose constraint by about 2.6 Gy. Although the maximum rectal dose constraint was not met in Case A at the PTV margins normally used at our institution, the rectal dose was lower than the usual clinical dose, so we adopted the usual clinical margins considering the effect of the setup. In Case B, the inserted SpaceOAR worked well and the rectal dose constraint was met. Since Case B showed that the rectal dose constraint could be met in cases where the SpaceOAR was successfully inserted, we have performed single irradiation in subsequent cases only when the SpaceOAR was accurately inserted and the rectal dose constraints used as reference were met.

Acute urinary tract adverse events such as increased frequency of urination and worsening urinary urgency, decreased urinary output, and nocturia were observed, but no late urinary tract adverse events such as urinary retention or hematuria were observed in any of the cases.

Although almost all dose constraints were met in the referenced study [3], Grade 3 late GI adverse events did occur. Therefore, there is still a possibility that a Grade 3 late adverse event may occur in the present case because the follow-up period for Cases B-D was less than one year.

Except for Case A, the rectal dose constraints were met. Consequently, there have been no Grade 3 or higher gastrointestinal adverse events during the current follow-up period. The use of a rectal balloon to suppress prostate motion did not appear to be necessary but could be replaced by the use of a SpaceOAR or additional margins. In all cases, PSA values at three months after irradiation showed a decrease, and the patients have since progressed without PSA recurrence.

## Conclusions

The risk of adverse events is higher with a single dose of stereotactic radiotherapy compared to hypofractionated irradiation. However, it was possible to irradiate with dose constraints met by appropriately inserting SpaceOAR.

In addition, the results suggest the potential for good disease control and safety of a single 24 Gy SBRT for prostate cancer. However, more patients and long-term studies are needed because of the small number of patients and the short follow-up period being used in this study.
